# The diagnosis and initial management of melanoma in Australia: findings from the prospective, population‐based QSkin study

**DOI:** 10.5694/mja2.51919

**Published:** 2023-04-11

**Authors:** Nirmala Pandeya, Catherine M Olsen, Maja M Shalit, Jean Claude Dusingize, Rachel E Neale, David C Whiteman

**Affiliations:** ^1^ QIMR Berghofer Medical Research Institute Brisbane QLD

**Keywords:** Melanoma, General practice, Diagnostic tests and procedures

## Abstract

**Objectives:**

To determine the proportions of newly diagnosed melanomas treated by different medical specialist types, to describe the types of excisions performed, and to investigate factors associated with treating practitioner specialty and excision type.

**Design, setting:**

Prospective cohort study; analysis of linked data: baseline surveys, hospital, pathology, Queensland Cancer Register, and Medical Benefits Schedule databases.

**Participants:**

Random sample of 43 764 Queensland residents aged 40–69 years recruited during 2011, with initial diagnoses of *in situ* or invasive melanoma diagnosed to 31 December 2019.

**Main outcome measures:**

Treating practitioner type and treatment modality for first incident melanoma; second and subsequent treatment events for the primary melanoma.

**Results:**

During a median follow‐up of 8.4 years (interquartile range, 8.3–8.8 years), 1683 eligible participants (720 women, 963 men) developed at least one primary melanoma (*in situ* melanoma, 1125; invasive melanoma, 558), 1296 of which (77.1%) were initially managed in primary care; 248 were diagnosed by dermatologists (14.8%), 83 by plastic surgeons (4.9%), 43 by general surgeons (2.6%), and ten by other specialists (0.6%). The most frequent initial procedures leading to histologically confirmed melanoma diagnosis were first excision (854, 50.7%), shave biopsy (549, 32.6%), and punch biopsy (178, 10.6%); 1339 melanomas (79.6%) required two procedures, 187 (11.1%) three. Larger proportions of melanomas diagnosed by dermatologists (87%) or plastic surgeons (71%) were in people living in urban areas than of those diagnosed in primary care (63%); larger proportions of melanomas diagnosed by dermatologists or plastic surgeons than of those diagnosed in primary care were in people with university degrees (45%, 42% v 23%) or upper quartile clinical risk scores (63%, 59% v 47%).

**Conclusions:**

Most incident melanomas in Queensland are diagnosed in primary care, and nearly half are initially managed by partial excision (shave or punch biopsy). Second or third, wider excisions are undertaken in about 90% of cases.

**The known:** Primary cutaneous melanoma is common in Australia, but little is known about how incident cases are initially managed.

**The new:** More than 75% of primary melanomas in a large Queensland sample of people aged 40–69 years were diagnosed and managed in primary care. About half were managed by excisional biopsy at first presentation, 33% underwent shave biopsy, and 10% were initially managed by punch biopsy. Almost 80% required a second, wider excision, and 11% required three procedures.

**The implications:** Most incident melanomas are managed by primary care practitioners, underscoring the need for specific training in this important area of medical practice.

Melanoma is an important public health problem in Australia, and its incidence^1^ and treatment costs are rising,^2^ particularly for advanced stage disease. The estimated lifetime risk of at least one melanoma diagnosis in Australia is 6.7% (one in fifteen).^3^ Primary care practitioners diagnose and manage the vast majority of keratinocyte cancers in Australia^4^ (in contrast to other countries^5^), but little has been published on how cutaneous melanoma is first diagnosed and which practitioners make the diagnosis.

Australian clinical guidelines recommend that people who present with suspicious pigmented lesions be promptly assessed by a primary care practitioner, by undertaking an excisional biopsy or referring them to a specialist.^6^, ^7^, ^8^ Primary care practitioners are therefore at the forefront of melanoma care, but information regarding diagnosis and treatment patterns in Australia is limited. Medical Benefits Schedule (MBS) reimbursement data indicate that 49% of melanomas diagnosed during 2013–14 had been excised by general practitioners, 34% by surgeons, and 16% by dermatologists.^9^ This analysis, however, was restricted to reimbursement claims for excisions of definitive melanoma, and did not include other procedures (such as shave or punch biopsy) often employed by Australian practitioners to establish a diagnosis of melanoma.^10^

Other Australian studies have described treatment patterns in highly selected samples of people with melanoma. A 2015 study^11^ examined patterns of surgical management for a series of Queensland people diagnosed with primary cutaneous melanoma at high risk of spread (clinical stages 1b or 2, comprising about 20% of all melanomas in Queensland); the authors reported that 80% of cases had been initially diagnosed by primary care practitioners (9% by surgeons, 10% by dermatologists), but definitive treatment was typically undertaken by surgeons. A 2007 study^12^ of invasive melanoma cases reported to the Victorian Cancer Registry found a substantially lower proportion of excisions by primary care practitioners (6.2%). However, the study sample was enriched for thicker melanomas, whereas more than 60% of invasive melanomas in Australia are less than 1 mm thick at diagnosis.^13^

Knowledge about how patients with melanoma are first managed, and which procedures are used to establish the diagnosis, is consequently incomplete. This knowledge is important, as the successful adoption of clinical guidelines requires their dissemination and educational activities directed at the practitioners expected to implement them. We therefore analysed linked data for a large prospective cohort of Queensland adults to determine the proportion of melanomas treated by different medical specialists, according to patient and tumour characteristics, to describe the types of excisions performed, and to investigate factors associated with the treating practitioner specialty and type of excision.

## Methods

The QSkin Sun and Health Study is a prospective cohort study of 43 764 men and women aged 40–69 years at recruitment, randomly sampled from the Queensland population in 2010–11.^14^ All participants consented to linkage of their data with health databases (including hospital, pathology, and cancer registry records) at baseline, and 92% consented to linkage with MBS reimbursement claims data (for medical services and consultations provided outside public hospitals to Australian citizens and permanent residents; http://medicarestatistics.humanservices.gov.au/statistics/mbs_item.jsp). Participants completed a baseline survey (https://www.qimrberghofer.edu.au/wp‐content/uploads/2020/08/QSkinStudyDocuments_V11_Nov2010‐1.pdf) that collected information on demographic characteristics, pigmentary and phenotypic characteristics, sun exposure and sun protection, family history of melanoma, past history of skin cancer excisions and other treatments for skin lesions, and history of skin examinations. The repeatability and validity of these items has been reported.^15^, ^16^ A melanoma risk prediction scoring tool has been developed and validated for the QSkin cohort.^17^

We restricted our analyses to participants for whom an incident *in situ* or invasive melanoma diagnosed between enrolment in 2010–11 and 31 December 2019 had been notified to the Queensland Cancer Register (QCR). Notification of melanoma diagnoses is a statutory requirement in Queensland. The registry records the date of diagnosis, anatomic site, morphology, and, for invasive melanoma, thickness, as well as the notifying practitioner. We further restricted our analyses to people who consented to record linkage with the MBS claims database, and excluded people with melanoma diagnoses prior to the baseline date.

### Treating practitioner and excision type

We obtained MBS claims data from the date of participant enrolment in 2010–11 to 31 December 2019 through data linkage conducted by Services Australia. We ascertained the type of treating practitioner for incident melanomas in the MBS claims data. To identify the first episode of care for melanoma for an individual in the MBS claims dataset, we searched for any claims for anatomical pathology services within ninety days of the date of diagnosis recorded by the QCR. We then searched for any claims related to skin biopsy, excision, or curettage (Supporting Information, tables 1 and 2) within the same period; the first such claim was designated the index event, and we recorded the practitioner type (primary care practitioner, dermatologist, plastic surgeon, general surgeon, other specialist). We then searched for codes related to follow‐up episodes of care (ie, second or wider excisions). For the 275 people who developed more than one primary melanoma during the follow‐up period, we restricted this analysis to episodes of care for their first melanoma. If no relevant services were recorded in the MBS claims data, we extracted the treating practitioner type from the pathology report supplied to the QCR.

The type of excision for an incident melanoma (both initial treatment and subsequent events for the same primary lesion) was recorded following manual review of QCR pathology reports as shave biopsy, punch biopsy, first excision, wide excision following an earlier procedure, other procedure, or not stated/unclear.

### Statistical analysis

Patient and tumour characteristics are reported as proportions, means (with standard deviations, SDs) or medians (with interquartile ranges, IQRs), and the statistical significance of between‐group differences was assessed in Pearson χ^2^ tests (categorical variables) or median score tests (continuous variables) in SAS 9.4. To estimate the association between practitioner type and the frequency of shave or punch biopsies relative to definitive excision, we calculated adjusted prevalence ratios (aPRs) with 95% confidence intervals (CIs) using log multinomial regression in Stata 8.0.^18^ *P* < 0.05 was deemed statistically significant.

### Ethics approval

The study was approved by the QIMR Berghofer Medical Research Institute human research ethics committee (P1309).

## Results

During a median follow‐up of 8.4 years (IQR, 8.3–8.8 years), 1697 participants without melanomas at baseline and who provided consent for MBS data linkage developed at least one primary melanoma. Nine participants were excluded because their initial notification to the cancer registry was for metastatic melanoma; five were excluded because information regarding neither provider nor treatment modality was available (ie, no MBS item codes and no pathology records identified). Our analysis therefore included 1683 people (720 women, 963 men) with primary cutaneous melanoma (*in situ* melanoma 1125; invasive melanoma, 558; Box 1). We identified a relevant MBS item code for the same date as the cancer registry notification for 1436 people (85%), for a further 133 within seven days (8%), and for 46 within thirty days (3%). We found no relevant MBS item claims linked with 68 melanoma diagnoses (4%), and for these cases we abstracted information about the referring doctor from the cancer registry pathology report.

Box 1 **Selection of QSkin participants with first incident invasive or *in situ* melanoma for our analysis**

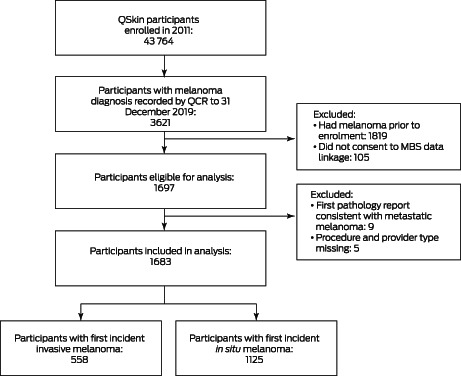

A total of 1296 melanomas were diagnosed following services by primary care practitioners (77.1%); 248 were diagnosed by dermatologists (14.8%), 83 by plastic surgeons (4.9%), 43 by general surgeons (2.6%), and ten by other specialists (0.6%) (Box 2; Supporting Information, figure 1). The most frequent initial procedures leading to histologically confirmed melanoma diagnosis were first excision (854, 50.7%), shave biopsy (549, 32.6%), and punch biopsy (178, 10.6%); for 44 melanomas (2.6%), the definitive diagnosis followed an earlier (presumably inconclusive) procedure (Box 2).

Box 2 **First incident melanomas diagnosed in 1683 eligible QSkin participants, Queensland, 2011–2019: diagnosing medical practitioner and treatment modality for the index treatment event**

**Table:** 

Treatment modality	All practitioners^*^	Primary care practitioner	Dermatologist	Plastic surgeon	General surgeon	Other specialist	*P* ^†^
Total number of people	1683	1296 [77.1%]	248 [14.8%]	83 [4.9%]	43 [2.6%]	10 [0.6%]	< 0.001
Shave biopsy	549 (32.6%)	388 (29.9%)	140 (56.5%)	13 (16%)	4 (9%)	2 (20%)	< 0.001
Punch biopsy	178 (10.6%)	159 (12.3%)	10 (4.0%)	5 (6%)	4 (9%)	0	< 0.001
First excision	854 (50.7%)	703 (54.2%)	67 (27%)	52 (63%)	25 (58%)	6 (60%)	< 0.001
Second (wide) excision	44 (2.6%)	20 (1.5%)	7 (3%)	10 (12%)	7 (16%)	0	0.016
Other^‡^	58 (3.5%)	26 (2.0%)	24 (10%)	3 (4%)	3 (7%)	2 (20%)	< 0.001

* Missing data (practitioner): shave biopsy, two; first excision, one.

† χ^2^ test for equal proportions (one‐way, practitioner type by treatment modality), except for the top row (two‐way, distribution of treatment modalities by provider type).

‡ Curettage, six; multiple procedure types (eg. shave and punch) listed in the pathology report, four; sampling procedure not specified in the pathology report, 38.

A total of 1339 melanomas (79.6%) were managed with two procedures, 187 (11.1%) with three. Second and third treatment episodes all involved wide excisions; 1067 second excisions (80.0%) and 161 third excisions (86.1%) were performed by primary care practitioners. Dermatologists used shave biopsy for initial management more frequently than other practitioners (Box 2; Box 3).

Box 3 **First incident melanomas diagnosed in 1683 eligible QSkin participants, Queensland, 2011–2019: treatment modality, by medical practitioner type**

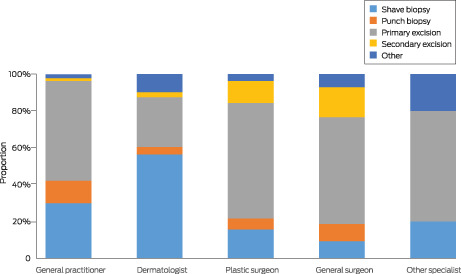

After adjustment for age, sex, and residential remoteness of the patient and anatomic site of the melanoma, dermatologists performed shave biopsy more frequently than primary care practitioners (aPR, 1.81; 95% CI, 1.59–2.06) and punch biopsy less frequently (aPR, 0.35; 95% CI, 0.19–0.65). Surgeons less frequently performed shave (aPR, 0.55; 95% CI, 0.37–0.81) and punch biopsy (aPR, 0.59; 95% CI, 0.31–1.12) than primary care practitioners.

More than half the primary melanomas diagnosed and managed by plastic surgeons were head and neck tumours (50.6%), compared with 19.3% of those diagnosed by other practitioners. Most invasive melanomas were thin (≤ 1.0 mm; 452 of 550, 82.2%); eighteen were thicker than 4 mm (3.3%). Of those diagnosed by dermatologists, 93% were thin melanomas, compared with 80.6% of those diagnosed by other practitioners (Box 4).

Box 4 **First incident melanomas diagnosed in 1683 eligible QSkin participants, Queensland, 2011–2019: tumour characteristics, by treating practitioner**

**Table:** 

Tumour characteristics*	Total	Primary care practitioner	Dermatologist	Plastic surgeon	General surgeon	Other specialist	*P* ^†^
Total number	1683	1296	248	83	43	10	0.030
*In situ* melanoma	1125 (66.8%)	872 (67.3%)	173 (69.8%)	50 (60%)	20 (46%)	7 (70%)	
Invasive melanoma	558 (33.2%)	424 (32.7%)	75 (30%)	33 (40%)	23 (54%)	3 (30%)	
**All melanoma**							
Body site							< 0.001
Head/neck	351 (20.9%)	246 (19.0%)	52 (21%)	42 (50.6%)	8 (19%)	3 (30%)	
Trunk	631 (37.5%)	520 (40.1%)	85 (34%)	9 (10.8%)	11 (26%)	3 (30%)	
Upper limbs	439 (26.1%)	335 (25.9%)	69 (28%)	18 (21.7%)	14 (33%)	3 (30%)	
Lower limbs	259 (15.4%)	194 (15.0%)	41 (16%)	14 (16.9%)	9 (21%)	1 (10%)	
Overlapping/not otherwise specified	3 (0.2%)	1 (0.1%)	1 (0.4%)	0	1 (2%)	0	
**Invasive melanoma**							
Histologic subtype							0.23
Superficial spreading	329 (60.0%)	251 (59.2%)	47 (62.7%)	20 (60.6%)	10 (44%)	1 (33%)	
Lentigo maligna	45 (8.1%)	29 (6.8%)	10 (13%)	3 (9%)	2 (9%)	1 (33%)	
Nodular	35 (6.3%)	28 (6.6%)	1 (1%)	4 (12%)	2 (9%)	0	
Other/not otherwise specified	149 (26.7%)	116 (27.4%)	17 (23%)	6 (18%)	9 (39%)	1 (33%)	
Lesion thickness (mm)							0.28
≤ 1.0	452 (82.2%)	345 (81.4%)	66 (93%)	24 (75%)	15 (71%)	2 (100%)	
1.01–1.99	54 (9.8%)	44 (10%)	4 (6%)	3 (9%)	3 (14%)	0	
2.01–3.99	26 (4.7%)	22 (5.2%)	1 (4%)	2 (6%)	1 (5%)	0	
≥ 4.00	18 (3.3%)	13 (3.1%)	0	3 (9.4%)	2 (10%)	0	
Missing data	8	0	4	1	2	1	
Mean (SD)	0.85 (1.23)	0.87 (1.24)	0.45 (0.33)	1.27 (1.89)	1.14 (1.26)	0.41 (0.13)	
Median (IQR)	0.50 (0.30–0.83)	0.50 (0.30–0.90)	0.35 (0.25–0.50)	0.50 (0.30–1.03)	0.60 (0.48–1.20)	0.41 (0.32–0.50)	< 0.001

IQR = interquartile range; SD = standard deviation.

* Missing data (practitioner): *in situ* melanoma, three; all melanoma/trunk as site, three; lesion thickness, eight.

† χ^2^ test (categorical variables) or median test (continuous variables).

Larger proportions of melanomas diagnosed by dermatologists or plastic surgeons than of those diagnosed in primary care were in people with university degrees (45%, 42% *v* 23%), private health insurance (90%, 87% *v* 70%), or upper quartile clinical risk scores (63%, 59% *v* 47%); larger proportions of people in these two groups also reported previous excisions for skin cancer (72%, 68% *v* 58%) or treatment for skin lesions (82%, 80% *v* 73%). Larger proportions of melanomas diagnosed by dermatologists (87%) or plastic surgeons (71%) were in people living in urban areas than of those diagnosed in primary care (63%) (Supporting Information, table 3).

A larger proportion of melanomas diagnosed by punch biopsy were invasive (80 of 178, 45%) than of those diagnosed by shave biopsy (139 of 549, 25.3%) or first excision (305 of 854, 35.7%). A larger proportion of invasive melanomas diagnosed by shave biopsy were thin (126 of 138, 91.3%) than of those diagnosed by other procedures (60–80%) (Box 5). A greater proportion of melanomas diagnosed by first excision were in men (516 of 854, 60.4%) than those diagnosed by other procedures (447 of 829, 53.9%). Finally, a larger proportion of melanomas diagnosed by shave biopsy were in people living in urban areas (428 of 549, 78.0%) than those diagnosed by first excision (496 of 854, 58.1%) (Supporting Information, table 4).

Box 5 **First incident melanomas diagnosed in 1683 eligible QSkin participants, Queensland, 2011–2019: treatment modality for the first tissue sample, by tumour characteristic**

**Table:** 

Tumour characteristics	Shave biopsy	Punch biopsy	First excision	Second wide excision	Other	*P* *
Total number	549	178	854	44	58	
*In situ* melanoma	410 (74.7%)	98 (55%)	549 (64%)	34 (77%)	34 (59%)	
Invasive melanoma	139 (25.3%)	80 (45%)	305 (35.7%)	10 (23%)	24 (41%)	< 0.001
Age at diagnosis (years), mean (SD)	63.1 (7.9)	63.7 (7.5)	62.6 (8.2)	62.0 (7.5)	61.6 (7.7)	
Age at diagnosis (years), median (IQR)	64.0 (57.6–69.2)	63.9 (58.8–69.9)	63.5 (57.5–69.0)	62.8 (56.5–67.4)	63.6 (56.6–67.8)	0.91
**All melanoma**						
Body site						
Head/neck	125 (22.8%)	37 (21%)	153 (17.9%)	17 (39%)	19 (33%)	
Trunk	179 (32.6%)	56 (32%)	365 (42.7%)	15 (34%)	16 (28%)	
Upper limbs	140 (25.5%)	45 (25%)	228 (26.7%)	10 (23%)	16 (28%)	
Lower limbs	105 (19.1%)	40 (22%)	107 (12.5%)	2 (5%)	5 (9%)	
Overlapping/not otherwise specified	0	0	1 (0.1%)	0	2 (4%)	< 0.001
**Invasive melanoma**						
Histologic subtype						
Superficial spreading	80 (58%)	45 (56.3%)	191 (62.6%)	4 (40%)	9 (38%)	
Lentigo maligna	14 (10%)	11 (14%)	18 (5.9%)	0	2 (8%)	
Nodular	5 (4%)	5 (6%)	22 (7.2%)	2 (20%)	1 (4%)	
Other/not otherwise specified	40 (29%)	19 (24%)	74 (24%)	4 (40%)	12 (50%)	0.041
Thickness (mm)						
≤ 1.0	126 (91.3%)	64 (80%)	244 (80.0%)	6 (60%)	12 (71%)	
1.01–1.99	8 (6%)	12 (15%)	31 (10%)	1 (10%)	2 (12%)	
2.01–3.99	4 (3%)	3 (4%)	18 (5.9%)	0	1 (6%)	
≥ 4.00	0	1 (1%)	12 (3.9%)	3 (30%)	2 (12%)	< 0.001
Missing data	1	0	0	0	7	
Mean (SD)	0.52 (0.47)	0.75 (0.73)	0.95 (1.40)	2.28 (2.76)	1.29 (1.66)	
Median (IQR)	0.40 (0.25–0.60)	0.50 (0.31–0.95)	0.54 (0.35–0.90)	0.89 (0.50–4.40)	0.75 (0.39–1.20)	< 0.001

IQR = interquartile range; SD = standard deviation.

* χ^2^ test (categorical variables) or median test (continuous variables).

## Discussion

We report the patterns of initial management for people newly diagnosed with melanoma in a large prospective cohort of Queensland adults. More than 75% of melanomas were initially managed in primary care, consistent with the high proportion of registered medical practitioners in Queensland who are general practitioners (7365 of 17 115, 43.0%; dermatologists: 117, 0.7%; general surgeons: 400, 2.3%; plastic surgeons, 82, 0.5%).^19^ The medical specialties differed in the types of excisions undertaken for initial melanoma management, and in the casemix of patients and tumours they managed. The proportions of people managed by dermatologists and plastic surgeons who were university‐educated or lived in urban areas were larger than those managed in primary care, which provided care for 73.2% of patients in urban areas and 84.5% of those in rural or remote areas. Plastic surgeons managed only 12% of people with head and neck melanomas, but these cases comprised 51% of the incident melanomas they managed.

Our finding that most new melanomas in Queensland are managed in primary care is consistent with an earlier analysis of MBS claims data that excluded shave and punch biopsy procedures.^9^ The authors found that definitive excision was undertaken by primary care practitioners in 49% of cases in Australia during 2013–14 (62% in Queensland), 34% by surgeons (Queensland, 29%), and 16% by dermatologists (Queensland, 9%). Our study differed in that we sought to identify the first treatment event that led to a histological diagnosis of melanoma (not just the definitive treatment), so that we also included melanomas diagnosed by partial excision (ie, shave or punch biopsy) as well as those diagnosed after surgical excision. Moreover, we confirmed each primary melanoma diagnosis and obtained the type of excision by referring to the corresponding QCR pathology report, reducing the potential for misclassification that could arise by relying on MBS reimbursement data alone. Our findings are also consistent with a recent analysis of Skin Cancer Audit Research Database (SCARD) data which found that 55.9% of melanomas diagnosed in primary care were diagnosed after elliptical excision biopsy, with 74.9% undergoing wide re‐excision by the treating general practitioner.^20^

While we found that the most frequent initial procedure was surgical excision, almost half of all melanomas were diagnosed following partial excision (shave or punch biopsy). Australian clinical practice guidelines for melanoma^8^ recommend that skin lesions suspected to be melanoma be completely excised by excisional biopsy with a 2 mm margin. The guidelines note that deep shave excision and punch excision methods can also be used, but that these approaches are more frequently associated with positive margins than elliptical excision and primary closure.^10^, ^21^ In our series, we found that 90% of people underwent two or more surgical procedures in the course of melanoma management, consistent with clinical practice guidelines that recommend an initial complete excisional biopsy, and subsequent re‐excision with a wider margin if indicated by information obtained from the excision biopsy (eg, invasiveness, Breslow thickness for invasive tumours).^8^ Most melanomas in our cohort (1577 of 1683, 94%) were *in situ* or thin (≤ 1.0 mm) invasive melanomas, which have an excellent prognosis; after a median of nine years’ follow‐up, we are aware of only seven deaths from melanoma in the cohort, suggesting that these tumours have been managed appropriately.

The growing burden of skin cancer in Australia requires efficient and effective use of health care resources. Our findings indicate that primary care practitioners play a major role in skin cancer treatment and management in Queensland, the Australian state with the highest incidence of melanoma, and where skin cancer clinics staffed by general practitioners are increasingly common.^22^, ^23^ However, MBS claims data indicate that the number of excisions to exclude melanoma is almost six times as high as the number for confirmed melanomas (440 019 *v* 70 435 in 2022), and the number of excisions for keratinocyte cancers dwarfs both categories (795 430 in 2022). We have previously reported that primary care practitioners also perform most keratinocyte excisions (83%).^4^ As demand for general practice services increases, it is worth reflecting that more than 65% of melanomas and almost 100% of keratinocyte cancers are preventable.^24^ Primary prevention therefore remains the key strategy for controlling skin cancer and reducing the future burden on primary care. General practitioners are perfectly positioned to provide prevention advice.

### Limitations

The strengths of our study include its prospective design, the population‐based sampling frame, and complete ascertainment of incident, histologically verified melanoma diagnoses (including melanoma *in situ*) through linkage with QCR data, cross‐referenced with MBS claims data (including second and subsequent episodes of care for the same tumour). Limitations included missing data on diagnostic procedures for 38 melanomas. While the baseline characteristics of the QSkin study cohort (23% of people invited to participate did so^14^) were similar to those of the general Queensland population, the mean age of participants (56 *v* 52 years) and the proportion of women (59% *v* 55%) were slightly higher than for people who declined to participate. Excluding the 5.8% of otherwise eligible participants from our analysis who did not consent to MBS data linkage may have influenced our findings, but the effect would be small.

### Conclusion

We found that many melanomas in Queensland are diagnosed after shave and punch biopsy, approaches not recommended by Australian guidelines as preferred modes for the initial management of suspicious pigmented lesions. Further research is needed to understand patterns of melanoma management in other parts of Australia, and to characterise the elements of care associated with optimal patient outcomes, providing insights that could inform revised melanoma management guidelines. In the meantime, our findings highlight the need to continue skin cancer training programs for primary care practitioners, who manage most patients with melanoma in Australia.

## Open access

Open access publishing facilitated by The University of Queensland, as part of the Wiley – The University of Queensland agreement via the Council of Australian University Librarians.

## Competing interests

No relevant disclosures.

## Supporting information


**Supporting Information**.
