# Supplementary material for: The diagnosis and initial management of melanoma in Australia: findings from the prospective, population‐based QSkin study
Source: Med J Aust. 2023 Apr 11;218(9):402–7. doi: 10.5694/mja2.51919 (PMC10953446; doi:10.5694/mja2.51919)
Supplement: Supplementary file 1 — Supporting Information. [file MJA2-218-402-s001.pdf]

The Medical Journal of Australia

Supporting Information

Supplementary methods and results

This appendix was part of the submitted manuscript and has been peer reviewed.
It is posted as supplied by the authors.

Appendix to: Pandeya N, Olsen CM, Shalit MM, et al. The diagnosis and initial management of melanoma in Australia: findings from the prospective, population-based QSkin study. *Med J Aust* 2023; doi: 10.5694/mja2.51919.

Table 1. MBS item numbers for the diagnosis and treatment of skin cancers and benign skin lesions, 2011-2015

	**Procedure**	**Item Numbers**	**Description**
SURGICAL EXCISION	Melanoma	31300<br>31305<br>31310<br>31315<br>31320<br>31325<br>31330<br>31335	nose, eyelid, lip, ear, digit or genitalia; ≤ 10 mm in diameter<br>nose, eyelid, lip, ear, digit or genitalia; > 10 mm in diameter<br>face, neck or lower leg; ≤ 10 mm in diameter<br>face, neck or lower leg; > 10 mm and ≤ 20 mm in diameter<br>face, neck or lower leg; > 20 mm in diameter<br>other areas of the body; ≤ 10 mm in diameter<br>other areas of the body; > 10 mm and ≤ 20 mm in diameter<br>other areas of the body; > 20 mm in diameter
	Benign lesions to exclude melanoma	31205<br>31210<br>31215<br>31220<br>31225<br>31230<br>31235<br>31240	single lesion; ≤ 10 mm in diameter<br>single lesion; > 10 mm and ≤ 20 mm in diameter<br>single lesion; > 20 mm in diameter<br>4-10 lesions; ≤ 10 mm in diameter<br>>10 lesions; ≤ 10 mm in diameter<br>nose, eyelid, lip, ear, digit or genitalia<br>face, neck or lower leg; ≤ 10 mm in diameter<br>face, neck or lower leg; ≤ 10 mm in diameter
	BCC/SCC, first surgical excision	31255<br>31260<br>31265<br>31270<br>31275<br>31280<br>31285<br>31290	nose, eyelid, lip, ear, digit or genitalia; ≤ 10 mm in diameter<br>nose, eyelid, lip, ear, digit or genitalia; > 10 mm in diameter<br>face, neck or lower leg; ≤ 10 mm in diameter<br>face, neck or lower leg; > 10 mm and ≤ 20 mm in diameter<br>face, neck or lower leg; > 20 mm in diameter<br>other areas of the body; ≤ 10 mm in diameter<br>other areas of the body; > 10 mm and ≤ 20 mm in diameter<br>other areas of the body; > 20 mm in diameter
	Residual and Recurrent BCC/SCC, previously treated surgically	31256, 31261, 31266, 31271, 31276, 31281, 31286, 31291, 31257, 31262, 31267, 31272, 31277, 31282, 31287, 31292, 31258, 31263, 31268, 31273, 31278, 31283, 31288, 31293	
	Residual and Recurrent BCC/SCC, previously treated non- surgically	31295	
	Moh surgery	31000<br>31001<br>31002	
BIOPSY	Biopsy	30071	
OTHER TREATMENT (cryotherapy or serial curettage)	Benign lesion	30195	
	Premalignant lesion	30192	
	Malignant lesion	30196, 30197, 30202, 30203, 30205	

BCC: Basal Cell Carcinoma; SCC: Squamous Cell Carcinoma.

Table 2. MBS item numbers for the diagnosis and treatment of skin cancers and benign skin lesions, 2016-2019

	**Procedure**	**Item Numbers**	**Description**
SURGICAL EXCISION	Melanoma	31371<br>31372<br><br><br>31373<br><br><br>31374<br>31375<br>31376	nose, eyelid, lip, ear, digit or genitalia; ≥6 mm in diameter<br>face, neck, scalp, nipple-areola complex, distal lower limb (distal to, and including, the knee) or distal upper limb (distal to, and including, the ulnar styloid); <14 mm in diameter<br>face, neck, scalp, nipple-areola complex, distal lower limb (distal to, and including, the knee) or distal upper limb (distal to, and including, the ulnar styloid); ≥14 mm in diameter<br>other areas of the body; <15mm<br>other areas of the body; 15-30 mm<br>other areas of the body; >30 mm in diameter
	Benign lesions to exclude melanoma	31357<br>31360<br>31362<br><br><br>31364<br><br><br>31366<br>31368<br>31370	nose, eyelid, lip, ear, digit or genitalia; <6 mm in diameter<br>nose, eyelid, lip, ear, digit or genitalia; ≥6 mm in diameter<br>face, neck, scalp, nipple-areola complex, distal lower limb (distal to, and including, the knee) or distal upper limb (distal to, and including, the ulnar styloid); <14 mm in diameter<br>face, neck, scalp, nipple-areola complex, distal lower limb (distal to, and including, the knee) or distal upper limb (distal to, and including, the ulnar styloid); <14 mm in diameter<br>other areas of the body; <15mm<br>other areas of the body; 15-30 mm<br>other areas of the body; >30 mm in diameter
	BCC/SCC, first surgical excision	31356<br>31358<br>31359<br><br>31361<br><br><br>31363<br><br><br>31365<br>31367<br>31369	nose, eyelid, lip, ear, digit or genitalia; <6 mm in diameter<br>nose, eyelid, lip, ear, digit or genitalia; ≥6 mm in diameter<br>nose, eyelid, lip, ear, digit or genitalia; excision area at least one third of surface area of the site<br>face, neck, scalp, nipple-areola complex, distal lower limb (distal to, and including, the knee) or distal upper limb (distal to, and including, the ulnar styloid); <14 mm in diameter<br>face, neck, scalp, nipple-areola complex, distal lower limb (distal to, and including, the knee) or distal upper limb (distal to, and including, the ulnar styloid); <14 mm in diameter<br>other areas of the body; <15mm<br>other areas of the body; 15-30 mm<br>other areas of the body; >30 mm in diameter
	Residual and recurrent BCC/SCC, previously treated surgically	31256, 31261, 31266, 31271, 31276, 31281, 31286, 31291, 31257, 31262, 31267, 31272, 31277, 31282, 31287, 31292, 31258, 31263, 31268, 31273, 31278, 31283, 31288, 31293	
	Residual and Recurrent BCC/SCC, previously treated non- surgically	31295	
	Moh surgery	31000<br>31001	

	Procedure	Item Numbers	Description
		31002	
BIOPSY	Biopsy	30071	
OTHER TREATMENT (cryotherapy or serial curettage)	Benign lesion	30195	
	Premalignant lesion	30192	
	Malignant lesion	30196, 30197, 30202, 30203, 30205	

BCC: Basal Cell Carcinoma; SCC: Squamous Cell Carcinoma.

Figure 1. Practitioner type for first incident melanoma

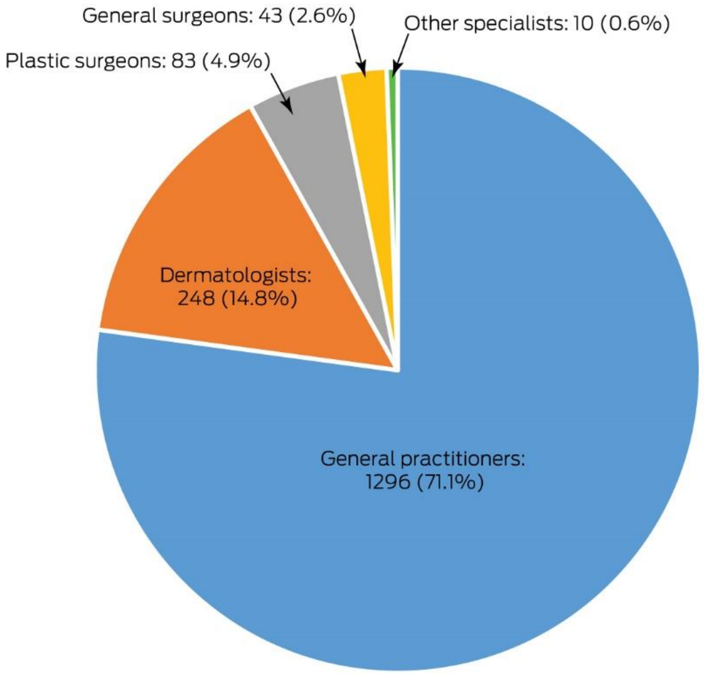

Table 3. Characteristics of all QSkin participants with an incident melanoma during follow-up, and according to treating practitioner for first excision.

Variable	Total	Primary care practitioner	Dermatologist	Plastic surgeon	General surgeon	Other specialist	Missing data	*P**
**Total number**	1683	1296	248	83	43	10		
**Age at baseline**								0.96
40-49	290 (17.2%)	219 (16.9%)	47 (19%)	15 (18%)	8 (19%)	1 (10%)	-	
50-59	617 (36.7%)	478 (36.9%)	92 (37%)	31 (37%)	13 (30%)	3 (30%)	-	
60+	776 (46.1%)	599 (46.2%)	109 (44%)	37 (45%)	22 (51%)	6 (60%)	3	
**Sex**								0.71
Women	720 (42.8%)	547 (42.2%)	113 (45.6)	33 (40%)	21 (49%)	5 (50%)	1	
Men	963 (57.2%)	749 (57.8%)	135 (54.4)	50 (60%)	22 (51%)	5 (50%)	2	
**Education**								<0.001
No school certificate	112 (7.2%)	99 (8.3%)	6 (3%)	5 (7%)	2 (5%)	0		
School certificate	543 (35.1%)	431 (36.2%)	67 (29%)	28 (37%)	14 (35%)	3 (33%)		
Trade/diploma	474 (30.6%)	387 (32.5%)	54 (23%)	11 (14%)	16 (40%)	5 (56%)	1	
University degree	419 (27.1%)	272 (22.9%)	105 (45%)	32 (42%)	8 (20%)	1 (11%)	1	
Missing	*135*	*107*	*16*	*7*	*3*	*1*		
**Private health insurance**								<0.001
No	444 (26.4%)	389 (30.1%)	26 (10%)	11 (13%)	14 (33%)	3 (30%)	1	
Yes	1236 (73.6%)	904 (69.9%)	222 (89.5%)	72 (87%)	29 (67%)	7 (70%)	2	
Missing	*3*	3	-	-	-	-		
**Region of residence**[1]								<0.001
Urban	1114 (66.2%)	815 (62.9%)	216 (87.1%)	59 (71%)	16 (38%)	7 (70%)	1	
Rural	556 (33.1%)	473 (36.5%)	28 (11%)	24 (29%)	26 (60%)	3 (30%)	2	
Remote	12 (0.7%)	7 (0.5%)	4 (2%)	0	1 (2%)	0	-	
Missing	*1*	*1*	-	-	-	-		
**Skin check in 3 years prior to baseline**								0.001
None	243 (14.6%)	204 (16.0%)	18 (7.3%)	11 (13%)	9 (21%)	1 (10%)		
1	419 (25.2%)	338 (26.5%)	49 (20%)	21 (26%)	9 (21%)	1 (10%)	1	
2 or more	1000 (60.2%)	734 (57.5%)	181 (73.0%)	50 (61%)	25 (58%)	8 (80%)	2	

Variable	Total	Primary care practitioner	Dermatologist	Plastic surgeon	General surgeon	Other specialist	Missing data	*P**
Missing	*21*	*20*	-	*1*	-			
**Clinical risk score**								0.001
Q1	115 (6.8%)	96 (7.4%)	14 (5.6%)	2 (2%)	3 (7%)	0		
Q2	264 (15.7%)	213 (16.4%)	29 (12%)	12 (14%)	7 (16%)	3 (30%)		
Q3	464 (27.6%)	378 (29.2%)	49 (20%)	20 (24%)	15 (35%)	0	2	
Q4	840 (49.9%)	609 (47.0%)	156 (63%)	49 (59%)	18 (42%)	7 (70%)	1	
**Family history of melanoma**								0.41
No	960 (65.5%)	735 (65.2%)	139 (62.6%)	54 (75%)	24 (69%)	5 (71%)	3	
Yes	506 (34.5%)	392 (34.8%)	83 (37%)	18 (25%)	11 (31%)	2 (29%)	-	
Missing	*217*	*171*	*26*	*11*	*8*	*3*		
**Past excisions for skin cancer**								0.002
None	658 (39.5%)	540 (42.1%)	70 (28%)	26 (32%)	16 (37%)	4 (40%)	2	
1	263 (15.8%)	206 (16.0%)	38 (15%)	12 (15%)	7 (16%)	0	-	
2+	747 (44.8%)	538 (41.9%)	138 (56%)	44 (54%)	20 (46%)	6 (60%)	1	
Missing	*15*	*13*	*2*	*1*	-			
**Past skin lesions destroyed**								0.010
None	421 (25.1%)	344 (26.7%)	44 (18%)	17 (20%)	13 (30%)	2 (20%)	1	
1-5	436 (26.0%)	345 (26.8%)	59 (24%)	16 (19%)	13 (30%)	3 (30%)	-	
6-20	445 (26.6%)	341 (26.5%)	68 (27%)	25 (30%)	8 (19%)	2 (20%)	1	
21+	373 (22.3%)	258 (20.0%)	77 (31%)	25 (30%)	9 (21%)	3 (30%)	1	
Missing	*8*	*8*	-	-	-			

* χ^2 test.

(1) Australian Bureau of Statistics. 1270.0.55.005. Australian Statistical Geography Standard (ASGS): Volume 5. Remoteness Structure, July 2011. 31 Jan 2013. https://www.abs.gov.au/ausstats/abs@.nsf/Lookup/1270.0.55.005main+features1july+2011 (viewed Dec 2022).

Table 4. Treatment modality for the first tissue sample of the first incident melanoma according to patient characteristics

Variables	Shave biopsy	Punch biopsy	First excision	Second wide excision	Other	*P**
**Total number**	549	178	854	44	58	
**Age at baseline**						0.93
40-49	99 (18%)	27 (15%)	148 (17%)	9 (20%)	7 (12%)	
50-59	193 (35.2%)	70 (39%)	314 (37%)	17 (39%)	23 (40%)	
60+	257 (46.8%)	81 (46%)	392 (46%)	18 (41%)	28 (48%)	
**Sex**						0.012
Women	238 (43.4%)	90 (51%)	338 (40%)	25 (57%)	29 (50%)	
Men	311 (56.6%)	88 (49%)	516 (60%)	19 (43%)	29 (50%)	
**Education**						0.45
No school certificate	35 (6.9%)	12 (7.3%)	60 (7.7%)	2 (5%)	3 (6%)	
School certificate	161 (31.9%)	67 (41%)	286 (36.5%)	12 (29%)	17 (32%)	
Trade/diploma	154 (30.5%)	44 (27%)	244 (31.2%)	16 (38%)	16 (30%)	
University degree	155 (30.7%)	41 (25%)	193 (24.6%)	12 (29%)	18 (33%)	
Missing	*44*	*14*	*71*	*2*	*4*	
**Private health insurance**						0.18
No	136 (24.8%)	47 (26%)	242 (28.4%)	10 (23%)	9 (16%)	
Yes	412 (75.2%)	131 (73.6%)	610 (71.6%)	34 (77%)	49 (84%)	
Missing	*1*	-	*2*	-	-	
**Region of residence[1]**						<0.001
Urban	428 (78.0%)	118 (66.7%)	496 (58.1%)	30 (68%)	42 (72%)	
Rural	115 (20.9%)	59 (33%)	352 (41.2%)	14 (32%)	16 (28%)	
Remote	6 (1%)	0	6 (0.7%)	0	0	
Missing	-	*1*	-	-	-	
**Skin check in 3 years prior to baseline**						0.037
None	70 (13%)	27 (15%)	134 (15.8%)	6 (14%)	6 (11%)	
1	112 (20.7%)	55 (31%)	227 (26.8%)	12 (28%)	13 (23%)	
2 or more	358 (66.3%)	95 (54%)	485 (57.3%)	25 (58%)	37 (66%)	

Variables	Shave biopsy	Punch biopsy	First excision	Second wide excision	Other	*P**
Missing	*9*	*1*	*8*	*1*	*2*	
**Melanoma clinical risk prediction score**						0.97
Q1	35 (6.4%)	13 (7.3%)	60 (7.0%)	4 (9%)	3 (5%)	
Q2	87 (16%)	31 (17%)	126 (14.8%)	8 (18%)	12 (21%)	
Q3	160 (29.1%)	50 (28%)	229 (26.8%)	11 (25%)	14 (24%)	
Q4	267 (48.6%)	84 (47%)	439 (51.4%)	21 (48%)	29 (50%)	
**Family history of melanoma**						0.41
No	302 (63.7%)	103 (64%)	495 (66%)	29 (78%)	31 (65%)	
Yes	172 (36.3%)	59 (36%)	250 (34%)	8 (22%)	17 (35%)	
Missing	*75*	*16*	*109*	*7*	*10*	
**Past excisions for skin cancer**						0.20
None	202 (37.1%)	81 (46%)	340 (40%)	19 (43%)	16 (28%)	
1	85 (16%)	28 (16%)	136 (16%)	4 (9%)	10 (17%)	
2+	258 (47.3%)	66 (38%)	370 (44%)	21 (48%)	32 (55%)	
Missing	*4*	*3*	*8*	-	-	
**Past skin lesions destroyed**						0.90
None	126 (23.1%)	47 (27%)	228 (26.8%)	11 (25%)	9 (16%)	
1-5	142 (26.1%)	48 (27%)	217 (25.5%)	11 (25%)	18 (32%)	
6-20	153 (28.1%)	44 (25%)	222 (26.1%)	11 (25%)	15 (26%)	
21+	124 (22.8%)	38 (22%)	185 (21.7%)	11 (25%)	15 (26%)	
Missing	*4*	*1*	*2*	-	*1*	

* χ^2 test.

(1) Australian Bureau of Statistics. 1270.0.55.005. Australian Statistical Geography Standard (ASGS): Volume 5. Remoteness Structure, July 2011. 31 Jan 2013. https://www.abs.gov.au/ausstats/abs@.nsf/Lookup/1270.0.55.005main+features1july+2011 (viewed Dec 2022).
